# Deformation Characterization of Glass Fiber and Carbon Fiber-Reinforced 3D Printing Filaments Using Digital Image Correlation

**DOI:** 10.3390/polym17070934

**Published:** 2025-03-29

**Authors:** Vivien Nemes, Szabolcs Szalai, Brigitta Fruzsina Szívós, Mykola Sysyn, Dmytro Kurhan, Szabolcs Fischer

**Affiliations:** 1 Central Campus Győr, Széchenyi István University, H-9026 Győr, Hungary; nemes.vivien@sze.hu (V.N.); szalaisz@sze.hu (S.S.); szivos.brigitta.fruzsina@sze.hu (B.F.S.); 2 Vehicle Industry Research Center, Széchenyi István University, H-9026 Győr, Hungary; 3 Department of Planning and Design of Railway Infrastructure, Technical University Dresden, D-01069 Dresden, Germany; mykola.sysyn@tu-dresden.de; 4 Department of Transport Infrastructure, Ukrainian State University of Science and Technologies, UA-49005 Dnipro, Ukraine; d.m.kurhan@ust.edu.ua

**Keywords:** carbon composite, glass fiber-reinforced, deformation analysis, material properties, DIC, 3D printing, mechanical testing

## Abstract

The paper offers an in-depth deformation study of glass fiber-reinforced and carbon composite filaments of 3D printers. During the certification, the authors used DIC (Digital Image Correlation) as a full-field strain measurement technique to explore key material traits as a non-contact optical measurement method. The insights captured through the DIC technology enabled to better understand the localized strain distributions during the loading of these reinforced filaments. The paper analyzes the glass fiber and carbon fiber filaments used in 3D printing that are reinforced with these materials and are subjected to bending and compressive loading. The segment presents how loading affects the performance of reinforced filaments when varying such factors as the deposition patterns, layer orientation, and other process parameters. Different types and combinations of reinforcements and printing variables were tested, and the resulting dependencies of mechanical parameters and failure modes were established for each case. Key conclusions demonstrate that the mechanical behavior of both carbon- and glass fiber-reinforced filaments is strongly affected by the 3D printing parameters, particularly infill density, pattern, and build orientation. The application of Digital Image Correlation (DIC) allowed for a precise, full-field analysis of strain distribution and deformation behavior, offering new insights into the structural performance of fiber-reinforced 3D printed composites. The findings from the study provide guidance for the proper choice of filling material and the optimal parameters for the 3D printing process of models with high-performance indexes and seamless applications in the automotive and industrial manufacturing sectors.

## 1. Introduction

The rapid development of 3D printing has enabled the more efficient production of parts with more complex geometries, which can be used in various industrial applications [1,2]. FDM (Fused Deposition Modeling) technology is one of the most widely used 3D printing processes, which builds objects from thermoplastic polymer filaments such as PLA and PETG in layers [1,2,3]. Fiber-strengthened composites such as carbon and glass fibers are often used to optimize mechanical properties and improve printed structures’ strength, stiffness, and coefficient of thermal expansion [4].

As reviewed by Kohutiar et al. [5], the effectiveness of these reinforcements strongly depends on the manufacturing technology used. Layer orientation, print parameters, and the type of polymer matrix significantly influence the mechanical performance of the final composite [5].

The increasing demand for lightweight and high-performance materials has led to the widespread application of fiber-reinforced composites, where both natural and synthetic fibers—such as glass and carbon—play a crucial role in improving mechanical behavior under complex loading conditions [6]. Previous studies have shown that glass fiber composites exhibit excellent mechanical properties, particularly in terms of fatigue resistance and impact strength [7].

In materials science, the study of the mechanical behavior of materials and the effects of different loads is widely used, with particular emphasis on exploring the relationships between mechanical and geometrical compositional properties [8,9].

The importance of understanding the interplay between microstructure and mechanical performance in composite systems has been highlighted even in food-grade materials, such as restructured pimiento alginate–guar gels, where internal structuring significantly influenced texture and mechanical integrity [10].

Digital Image Correlation (DIC) is an effective solution for accurately assessing the mechanical performance of objects and can be used in a wide range of applications, whether in engineering, materials science, or electronics [11,12]. It provides a non-contact measurement procedure that allows a detailed analysis of the deformation and stress distribution of materials, giving a more accurate picture of the behavior of the structures under test [12,13].

Increasing industrial competitiveness, raw material efficiency, sustainability considerations, and the potential to develop complex products have made additive manufacturing (AM) technologies even more promising in recent years [1,2]. Additive manufacturing is a technology in which materials are built up layer by layer, allowing objects to be created from 3D model data [1,2]. This process is particularly advantageous for complex geometric shapes, although its drawbacks include limited surface quality and geometric accuracy [1,2].

3D printing is one part of additive manufacturing. There are many different 3D printing methods, the most common of which include Stereolithography (SLA), Selective Laser Sintering (SLS), Lamination Object Manufacturing (LOM), and Fused Deposition Modeling (FDM) [1,2].

FDM (Fused Deposition Modeling) is the most widely known 3D printing technology for industrial applications, mainly due to the low cost of printers and the wide range of fiber materials available at affordable prices. FDM 3D printers are often used in various industries such as automotive, aerospace, construction, and medical, especially in the field of rapid prototyping [1,2,3]. The printing material is a thermoplastic polymer, such as polylactic acid (PLA), acrylonitrile butadiene styrene (ABS), or polyamide (PA) filament [1,2,3].

The use of ABS filament, one of the most common materials in the world of 3D printing, is declining due to its disadvantages in printability.

The 8th PLA World Congress was held in Munich in 2024, during which it was said that PLA is a very advanced material, but it has functional and mechanical limitations. For this reason, the aim is to design and develop special PLA composites with increased heat resistance, printability, tensile strength, and impact strength compared to basic PLA materials with pseudo reinforcement [14].

**Figure 1:**
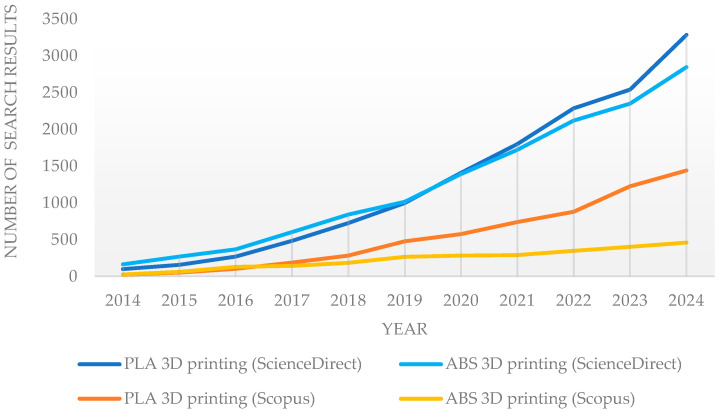
Number of search results for PLA and ABS 3D printing on ScienceDirect and Scopus databases.

The increase in the popularity of PLA is also reflected in the growing number of PLA 3D printing-related papers in research databases (ScienceDirect and Scopus), which overtake ABS, as illustrated in Figure 1.

From this point of view, it was considered important to investigate these dynamically evolving base materials, e.g., composite or carbon fiber-reinforced PLA matrix materials, to see what properties can be achieved with different reinforcements.

Conventional PETG was considered important to include in the materials to be tested because of its good impact resistance and toughness, which are superior to basic PLA, and its printability properties are better than ABS.

Due to the microstructural anisotropy and the layer-by-layer architecture of FDM technology, the resulting parts’ mechanical performance and manufacturing quality are generally inferior to those made using conventional manufacturing processes [15]. During FDM printing, the individual layers do not always adhere perfectly to each other, which can affect the strength and structural integrity of the final product, especially in the bonding planes between the layers [15].

Bembenek et al.’s [16] research shows that the printing angle directly affects Young’s modulus [16]. The results suggest that the printing direction plays a significant role in the final mechanical properties, and selecting the adequate angle can be a key factor in achieving the mechanical performance appropriate for the application [16].

However, pure polymers are unsuitable for printing structures where the printed object must have electrical conductivity or significant mechanical properties [1,4]. The solution is to add different reinforcements and fillers to the polymer matrixes, thereby increasing their structural stability and adding functional properties to the reinforcements [1,4].

Carbon, glass, and aradmite fibers are the most commonly used reinforcing fibers. These fibers can be continuous or discontinuous. Tests show that composites reinforced with continuous fibers have better mechanical properties than those reinforced with short fibers [4].

In addition to glass and carbon fibers, nanocellulose, as a natural reinforcing material, also presents a promising opportunity to enhance the mechanical properties of 3D printed composites [17].

The fiber-reinforced polymer filaments used for FDM 3D printing can be either short fiber-reinforced thermoplastic (SFRT) composites or continuous fiber-reinforced thermoplastic (CFRT) composites. In the case of continuous fiber embedding, embedding can occur before the printing process or directly in the print head [2].

For example, acrylonitrile butadiene styrene polymer (ABS) or polylactic acid (PLA) can be the basis for the matrix of FDM-printed continuous fiber composites (CF composites) [18]. Carbon fiber reinforcement is often used in PLA matrix, which increases mechanical strength by enabling excellent mechanical performance, and is lightweight and can be used in aerospace applications, including aircraft, spacecraft, and various engineering applications [1,3,4].

The application of composite materials in additive manufacturing is gaining increasing attention in both biomedical and industrial applications, as they optimize mechanical properties and provide a sustainable alternative to traditional polymers [19].

The results of Maqsood et al. [4] showed that the average flexural stress value of continuous carbon fiber-reinforced PLA (PLA-CCF) specimens is 103% higher than that of PLA specimens, whereas the average flexural stress value of short carbon fiber-reinforced PLA (PLA-SCF) specimens is 91.6% of that of PLA. In addition, it was found that PLA-CCF has the highest flexural modulus, but the flexural modulus of PLA-SCF also exceeds that of PLA [4].

In the research by Heidari–Rarani et al. [2], an experiment was conducted on a self-made continuous fiber-reinforced PLA composite, and the results showed that its maximum flexural strength increased by 109% and its flexural modulus by 367.6% compared to pure PLA [2].

Durga Prasada Rao et al. [20] used short carbon fiber-reinforced PLA filament for FDM 3D printing. By investigating two filling patterns, cubic and quarter cubic, the results showed that specimens fabricated with cubic patterns have high tensile strength values [20].

Based on the research of Giani et al. [18], it is known that polymer matrixes reinforced with carbon fiber reduce the coefficient of thermal expansion of the material and increase its thermal conductivity [18]. This results in printed objects with less warping and increased dimensional accuracy [18]. In addition, it has been found that PLA material with 10 wt% recycled carbon fiber, when 3D printed at 0° to the applied stress orientation, will exhibit approximately twice the elastic modulus and maximum fe-stress compared to the 90° orientation [18].

In addition to carbon fiber, glass fiber is a fiber reinforcement that can be used to increase the strength of PLA [21].

Glass fiber reinforcements dominate the composites industry, accounting for up to a significant percentage of all fiber reinforcements worldwide [21]. The thin surface coating applied during fiberglass production, known as sizing, significantly affects composite materials’ mechanical properties, durability, and manufacturability, reducing fiber breakage [21]. Film-forming materials such as polyvinyl acetates, polyurethanes, and epoxy resins provide protection and facilitate processing, while coupling agents such as APTES, GPTMS, and MPTMS improve fiber–matrix adhesion [21].

Wang et al. [22] investigated the extent to which glass fiber reinforcement can improve the inherently limited mechanical performance of PLA using silane-modified glass fibers (m-GF) blended with PLA at different wt% (5, 10, 15, and 20 wt%) [22]. It has been shown that with the addition of glass fiber, the tensile strength and stiffness increased almost linearly with increasing glass fiber content, and when 20 wt% GF was used, these values increased almost twofold compared to pure PLA [22]. The impact strength showed an even more visible increase: compared to 30.9 J/m for the base PLA, the impact resistance increased to 102.8 J/m for the composite containing 20 wt% GF, an improvement of more than three times [22]. In addition, the glass fibers were shown to limit thermal deformation but did not in themselves increase the thermal stability of PLA, yet positively affected its processability and foamability [22].

Begum et al. [23] investigated GF/PLA filaments printed in 0°, 45°, and 90° orientations at 40%, 50%, and 60% fill densities [23]. In the flexural test, the specimen with 60% fill density and 90° orientation showed the highest strength, which was 25 MPa, 60% higher than the specimen with 0° orientation [23]. At 90° orientation, the material can resist bending forces more effectively, distributing stress and improving the adhesion and structural integrity of the layer [23]. The material’s microstructure at 90° orientation allows for better deformation and energy dissipation under impact [23].

Liu et al. [24] investigated the mechanical properties of wood-, ceramic-, copper-, and carbon fiber PLA-based composites [24]. By FDM printing the specimens with different orientations and screen angles, they found that the specimens with edge-matched +45°/−45° screen angles exhibited the highest mechanical strength [24]. Due to the weak bonding between the layers, the vertically printed composite test specimens showed the weakest mechanical strength and modulus [24].

Comparing different fiber reinforcements, wood and carbon fiber-reinforced objects showed the lowest mechanical properties [24].

Ismail et al. [25] prepared composites using several methods. The methods involved the integration of composite fibers into thermoplastic matrixes; the methods differed in the techniques of integrating the fibers into the matrix (before printing, during extrusion, and during the printing process) [25]. Subsequently, different polylactic acid (PLA) and three different glass fiber-reinforced polylactic acid (GFPLA) composites were 3D printed with 1.02%, 2.39%, and 4.98% glass fiber content, respectively, and subjected to tensile tests [25]. The tensile strength of the composites increased from GFPLA-1 (1.02% glass fiber content) to GFPLA-2.4 (2.39% glass fiber content) but decreased dramatically at GFPLA-5 (4.98% glass fiber content), although it was still higher than that of pure PLA [25]. Finally, SEM studies showed that the porosity area decreased with increasing glass fiber content [25].

Research has shown that these properties improve proportionally with increasing fiber content, although fragility may also increase in individual cases, so it is important to find the optimum ratio [25].

The literature shows that adding carbon and glass fiber in the additive manufacturing of thermoplastics improves the mechanical properties of the product [26].

Goh et al. [27] tested objects made of carbon- and glass fiber-reinforced thermoplastic materials. From the results of the indentation test, it is concluded that glass fiber has a higher indentation resistance than carbon fiber [27]. It was also found that the typical fracture mode of additively manufactured carbon and glass fiber specimens is similar [27].

Alongside PLA, PETG is one of the materials most commonly used in 3D printing [28].

According to research by Mehtedi et al. [29], PETG is more flexible, tougher, less prone to fracture, and more resistant to external stresses compared to PLA [29].

The results of Martins et al. [30] also confirmed that PETG is a flexible material with high elongation and higher deformation capacity compared to PLA, which is more rigid and resistant to tensile forces. It can break more easily due to its rigidity [30].

Bembenek et al. [16] investigated FDM specimens, focusing on the infill pattern, and found that cubic infill for PLA and lines for PETG gave the best UTS (ultimate tensile strength) and STS (specific tensile strength) results [16].

Kadhum et al. [31] investigated the effect of 14 different filling patterns on the mechanical properties of 3D printed PLA and PETG objects with the same filling percentage [31]. Both materials’ highest tensile strength values occurred for gyroid and concentric designs. PETG showed higher strength when comparing the two polymers [31].

Srinivasan et al. [32] investigated the effect of filling patterns on the tensile strength of parts fabricated by FDM printing with PETG filament. The results showed that the grid infill pattern resulted in the highest tensile strength (36.34 MPa) compared to the other patterns [32]. The lowest tensile strength values (13.54 MPa) were obtained for the concentric infill specimen [32].

For 3D printing, the appropriate choice of filling pattern can optimize the object’s size and influence its strength, elasticity, and mechanical properties [33].

Guessasma et al. [33] developed a blood-element framework to investigate the effect of filling patterns and density and showed that honeycomb samples were the best choice for compression performance. It was shown that for the same fill ratio, among the gyroid, zigzag, and cross patterns, the gyroid pattern is the best option for improving mechanical strength, while the zigzag and cross are more suitable for promoting higher stretch–strain deformation, especially at low fill ratios [33].

Maqsood et al. [34] fabricated continuous carbon fiber-reinforced specimens with PLA matrix material using FDM 3D printing technology with grid and triangular infill patterns at 20%, 40%, and 60% infill densities. The grid specimen showed better mechanical properties than the triangular specimen. Based on the micrographs of the fractured composite, spallation was found to be the main cause of the failure [34].

The DIC system can be easily applied to various deformation tests, providing a versatile and reliable measurement technique for industrial and academic research [12].

In order to obtain a more accurate picture of material deformation during the Erichsen test (ECT, i.e., Erichsen Cupping Test), 3D Digital Image Correlation (DIC) can be used, which provides extensive information on stress and displacement distributions and fracture mechanisms and can calculate the mechanical properties of different materials [12].

The DIC technique is an efficient, non-contact, and highly accurate measurement method for Erichsen tests [12]. Research has shown that the DIC technique can be used to record stress distributions over the entire tested surface and identify localized thinning and material defects [12]. Compared to traditional single-point methods, this technique provides a more comprehensive picture of the material’s ductility [12]. The integration of DIC into ECT significantly improves the evaluation of deformation and fracture characteristics of metal plates [12].

The challenge of DIC, for example, in larger structures, is to optimize camera placement and dissection [35]. In addition, optical noise, lighting conditions, and camera stabilization can significantly impact the accuracy of measurements [35]. Damage or movement of speckle patterns can affect measurement accuracy, especially in fracture experiments [35].

It provides full area coverage measurements, which is a significant advantage over conventional sensors [35]. DIC can be used in concrete, steel, and composite structures, for example, to analyze the mechanical properties of complex structural elements such as composite materials [35].

Guo et al. [36] applied Digital Image Correlation (DIC) technology in tension–tension fatigue tests of carbon/glass fiber hybrid rods, using the method to map damage propagation, stress concentrations, and stiffness degradation. Their results confirm that DIC is an effective tool for tracking the deformation behavior of hybrid composites [36].

The executed literature review can be summarized below.

- Additive manufacturing is a technology that builds materials layer by layer from 3D model data, allowing complex objects to be created [1,2].
- Additive manufacturing is particularly advantageous for performing complex geometric shapes, although disadvantages include limited surface quality and geometric accuracy [1,2].
- Increasing industrial competitiveness, raw material efficiency, and sustainability are driving the growing importance of additive manufacturing technologies [1,2].
- 3D printing is one of the best-known forms of additive manufacturing, and it includes many processes such as SLA, SLS, LOM, and FDM [1,2].
- In industrial applications, FDM technology is the most common due to the low cost of printers and the wide availability of raw materials [1,2,3].
- FDM printers are used in various industries, such as automotive, aerospace, and medical, mainly for rapid prototyping [1,2,3].
- During FDM printing, the individual layers do not always adhere perfectly to each other, which can affect the mechanical properties of the final product [15].
- Research by Bembenek et al. [16] has shown that the printing angle directly influences Young’s modulus, so choosing the appropriate printing direction is key to optimizing mechanical performance [16].
- Pure polymers’ mechanical and electrical properties are not always satisfactory, so various reinforcements such as carbon, glass, and aramid fibers can be used to increase their strength and functional properties [1,4].
- Research shows that continuous fiber-reinforced composites have better mechanical properties than their short fiber-reinforced counterparts [4].
- Carbon fiber reinforcement in PLA matrix increases the strength of the material and reduces its thermal expansion coefficient, making it suitable for aerospace and engineering applications [1,3,4,18].
- Glass fiber reinforcement dominates the composites industry worldwide, improving mechanical strength and durability and reducing brittleness [21].
- Research by Wang et al. [22] has shown that fiberglass reinforcement increases tensile strength and impact resistance and limits thermal deformation [22].
- Studies by Begum et al. [23] have shown that the printing orientation and the filling density significantly influence the strength of 3D printed GF/PLA composites.
- Based on the research of Liu et al. [24] among different fiber reinforcements, samples with a raster angle of +45°/−45° showed the best mechanical performance, while composites printed in the vertical direction were the weakest [24].

While additive manufacturing has advanced considerably—particularly in the field of fiber-reinforced 3D printing—significant knowledge gaps remain regarding the mechanical behavior of these composite materials under different loading conditions. It is well established that adding carbon and glass fibers to polymer matrices enhances properties such as strength, stiffness, and impact resistance. However, most existing studies tend to examine isolated factors—such as fiber orientation, infill pattern, or interlayer adhesion—without fully considering how these parameters interact in practical, load-bearing applications. Furthermore, although Digital Image Correlation (DIC) is a widely adopted method for strain measurement, its potential to deliver high-resolution, full-field deformation data in the context of fiber-reinforced 3D printed components has yet to be fully exploited. The intrinsic anisotropy of FDM-manufactured parts, compounded by inconsistencies in layer bonding and filament deposition, adds complexity to predicting their mechanical performance. This study aims to address these challenges by leveraging DIC to perform a comprehensive, full-field analysis of the deformation and failure behavior of PLA- and PETG-based carbon- and glass fiber-reinforced 3D printing filaments.

The research systematically investigates the effects of printing parameters—including infill pattern, density, and build orientation—under both bending and compressive loads.

Through this approach, the study seeks to identify critical structure–property relationships that influence mechanical performance and to derive optimized printing strategies for enhanced material efficiency and functional reliability. The insights gained are intended to inform the design and manufacturing of high-performance, lightweight components in engineering applications where mechanical integrity and material efficiency are paramount.

The structure of the current paper is as follows. Section 2 contains the applied materials and methods, Section 3 introduces the results and gives explanations, and Section 4 provides the conclusions.

## 2. Materials and Methods

This section focuses on the materials and instruments used in the research and describes the measurement procedures and processes. The current study is based on three PLA-based filaments and one PETG filament from the Fillamentum, Filaticum, and eSun manufacturers.

### 2.1. Filaments

The four most common filaments available to the authors of the current study were selected for 3D printing the test specimens needed to perform the tests.

Fillamentum PLA is a conventional PLA that is easy to handle, has excellent print quality, and has excellent adhesion. Filaticum PETG is the ideal choice when a stable formulation and high heat resistance are required. Printed parts made from this material have the advantage of excellent resistance to chemical attack and retain their shape at temperatures up to 80 °C. The PLA-based filament eSUN ePLA with 15–20% glass fiber reinforcement is an easy-to-print filament. With glass fiber reinforcement, it exceeds conventional PLA’s mechanical strength and impact resistance. Pieces made from ePLA-GF have high impact strength, resistance, and abrasion resistance. eSUN ePLA-CF is a high-quality, easy-to-print PLA-based filament. Incorporating impregnated short carbon fibers increases the strength and modulus of PLA. It offers exceptional printing performance and high-speed printing. The main properties of all the used filaments are shown in Table 1. Table 2 summarizes the mechanical properties of the materials.

### 2.2. 3D Printers and Printing Setups

To print the test specimens, two reliable, easy-to-use, affordable and readily available FDM 3D printers were applied, which were selected for their ability to print the selected fiber-reinforced composites; their properties are shown in Table 3.

For the 3D prints, the easy-to-use Bambu Studio v1.9.7.52 slicing software for the Bambu Lab printers was used, which provides a wide range of options for adjustment. The fill percentage, fill pattern, and construction orientation were adjusted for the research.

**Figure 2:**
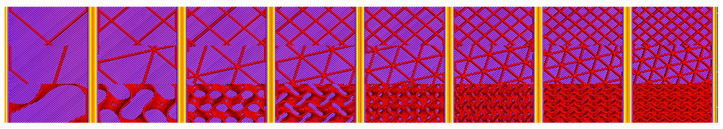
Filling settings in Bambu Studio for specimens in laid orientation; filling patterns from top to bottom: grid, triangle, gyroid; filling values from left to right: 5%–10%–15%–20%–25%–30%–35%–40%.

**Figure 3:**
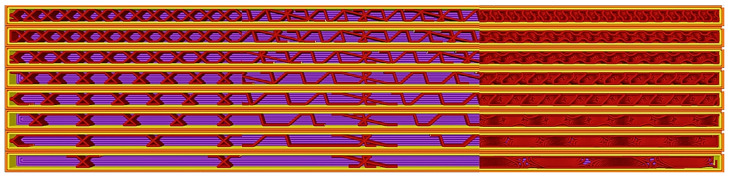
Filling settings in Bambu Studio for specimens with longitudinal edges; filling patterns from left to right: grid, triangle, gyroid; filling values from bottom to top: 5%–10%–15%–20%–25%–30%–35%–40%.

The density of the fill indicates the amount of material used for the fill. The fill values for this research were set between 5% and 40% with a 5% step size. The infill pattern represents the material’s structure within the 3D printed object, whereby the printed pieces can have different strength, elasticity properties, and bulk properties. One of the aims of this research is to investigate different patterns; the authors have investigated the most popular grid, triangle, and gyroid patterns for this research (Figure 2 and Figure 3).

**Figure 4:**
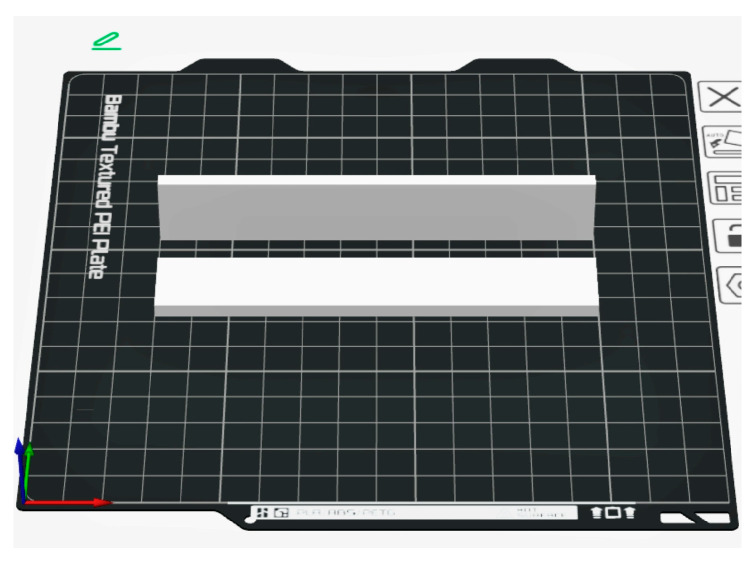
Demonstration of 3D printing orientations.

The test specimens were printed with two different construction orientations, laid flat and longitudinal edge-on (Figure 4). The orientation is important because the different ply alignments cause the forces through a ply to differ. The experimental matrix is presented in Table 4.

### 2.3. GOM Aramis DIC System

The measurement process was carried out using the GOM ARAMIS 5M non-contact, photogrammetric system. The GOM ARAMIS 2018 3D measurement system is part of the Digital Image Correlation (DIC) systems. The system allows the deformation of individual objects to be measured during the inspection. The principle of operation of the measurement system is based on photogrammetry. A sequence of photographs of the object, taken by two cameras in time-lapse mode, provides information on the 3D displacement of each point on the measured sample surface, independently of the material, by means of image correlation using random pattern recognition. The method can be used to inspect both small and large specimens [37,38,39,40].

The development of the approach for analyzing deformation behavior and structural integrity was supported by previous research, which demonstrated that the internal microstructural arrangement significantly impacts the mechanical properties and strength of materials [10].

The system uses two 12 MP cameras (GOM, Braunschweig, Germany), which enable high-resolution measurements, allowing small and large displacements to be detected [40].

### 2.4. Specimen Preparation and Speckle Pattern

**Figure 5:**
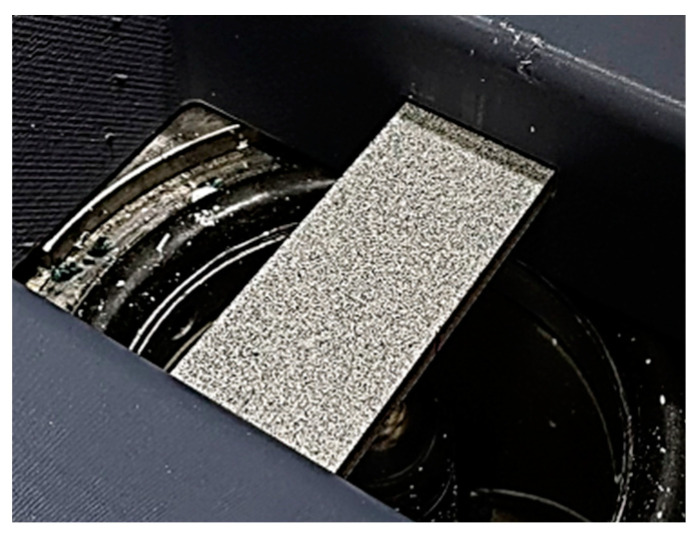
Speckle pattern on the specimen before the ECT.

The ARAMIS 3D measuring system (GOM, Braunschweig, Germany) determines the position of the points on the surface under test from a grey-scale image. Preparing the sample surface properly and using an adequate pattern is essential to obtain accurate measurement results. In order to achieve appropriate contrast and minimize reflections, the surface should first be coated with white matte paint. A gradient pattern of black spots (Figure 5) is then applied and distributed so that the ratio of white to black areas remains 50/50. The size and distribution density of the patches will depend on the size of the area under study. When measuring the deformation of small objects, the gradient should consist of finer, more densely spaced patches, whereas for larger objects, it is recommended to use sparser, larger patches. Pattern contrast, spot size, and distribution are key factors for accurate measurement [38,40].

The applied paint materials were Schuller Eh’klar PrismaColor Acryl RAL9016 (Schuller Eh’klar, Pécs, country: Hungary) for the white primer layer and United Sprays Matte Black Spray (United Paints and Chemicals S.A.E., Giza, Egypt) paint for the speckle pattern.

## 3. Results and Discussion

As can be seen in Table 4, 192 different combinations of tests could be performed, maintaining a repeatability of 3 per test, which means 576 tests per test (in ECT). Any conclusions or analyses presented in the results section are the average of the 3 measurements for ease of interpretation of the data.

The results are presented in two ways; the first is to conclude the conventional crack maximum and the force data measured at that time. The data presented here are derived from the hydraulic equipment’s travel and force display by the operator at the moment of crack initiation. Of course, the resulting data are presumably more inaccurate since they depend to a large extent on the operator’s speed, but trends can, of course, already be observed here. In the evaluation, the force-time and displacement-time data are mainly analyzed.

In the second case, the DIC tests were evaluated. The distance and forces are directly linked to the ARAMIS measurement card. In this case, no reading is performed by the operator, so this type of measurement result does not contain any reading uncertainty. The travel-time and displacement-time data are indirectly integrated into the measurement evaluation. In the evaluation, conclusions are drawn from these data. Thanks to the DIC evaluation, much data is also presented, and at the end of the Results chapter, the results of special or outlier measurements are analyzed.

### 3.1. Measurement Experience, Die Displacement Results

Table 5 shows the results of the tool displacement measurements. Several conclusions can be drawn from the analysis of the data. The PETG material shows the highest values overall, with the highest deformation measured. Additionally, in the case of PETG material, it can be observed that orientation is important, as specimens placed edge-on gave better results than those laid flat. This is interesting because, due to the layer order, the higher value would have been expected in the opposite case, presumably due to the effect of the filling. For the PLA material, the gyroid infill pattern gave the best results between 5 and 20%. Interestingly, this value decreases with increasing pattern density, while it increases with the other two patterns. This implies that good flexural strength results can be obtained for PLA material with a lower filling density, even with a gyroid filling pattern.

For the glass and carbon composites, the measurement results are stable, with no significant differences in filling rates or patterns. It is observed that the grid and triangle patterns showed similar performance for all materials. The effect of layout can significantly affect the results, especially for PETG and PLA materials. For glass and carbon materials, the values are relatively uniform and less sensitive to the fill pattern and ratio, which also indicates that using these materials can achieve similar mechanical properties at lower fill values, which can result in significant weight savings for a structure.

### 3.2. Results of DIC Measurements

The results of the DIC measurements are shown in diagram X. In such measurements, although the sub-applicant manufacturer has long been a leading automotive and research supplier, it is worthwhile to check, i.e., validate the results. Validation ensures that the data, models, or systems are reliable, accurate, and a good representation of reality, thus minimizing the risks of errors. By evaluating the displacement-time data of the Aramis system, the maximum displacement values were compared with the values in Table 4 for each material. It was found that the results of the Aramis system reproduce the values measured in the conventional measurement.

**Figure 6:**
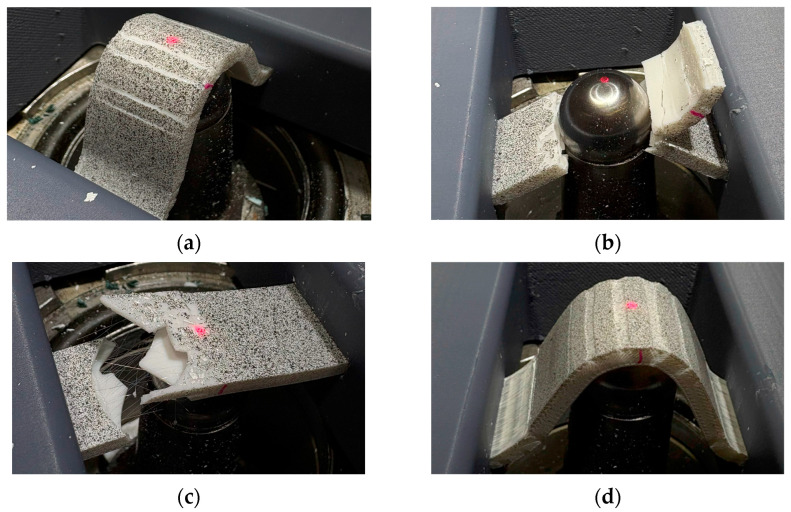
Different infill types in PETG specimens: (**a**) PETG_GrE40%, (**b**) PETG_GrE25%, (**c**) PETG_GrE5%, (**d**) PETG_TrE35%.

From the results in Figure 6, it is clear that the displacement results are also quite close. The results of the DIC measurements add a lot to the results of the conventional tests. If the slopes of the curves are almost identical, the following conclusion can be drawn. The curves’ steepness is related to the stiffness (k), which characterizes the resistance of the material or structure to deformation. If the slope is the same, then the stiffness of the specimens is the same, i.e., the initial resistance to bending of the materials and geometries tested is similar. The different displacement maxima show that, despite the same stiffness, the materials may behave differently at fracture due to internal structural differences. These differences are reflected in differences in infill density and pattern. The conclusions that can be drawn here are the same as in Section 3.1. The additional information provided by the DIC tests is that the whole forming process can be traced.

**Figure 7:**
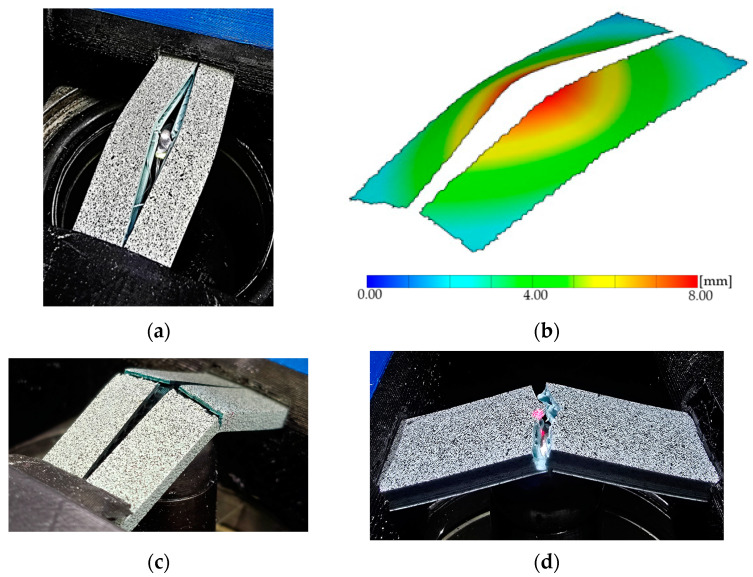
Different infill types in PLA specimens: (**a**) PLA_GrE5%, (**b**) PLA_GrE5% with DIC, (**c**) PLA_GrF15%, (**d**) PLA_GyF20%.

**Figure 8:**
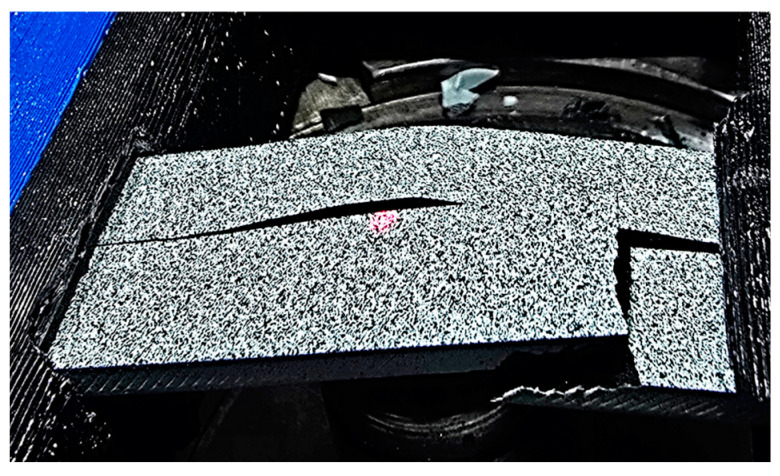
Brittle behavior in the carbon fiber specimen (i.e., CF_TrE25%).

**Figure 9:**
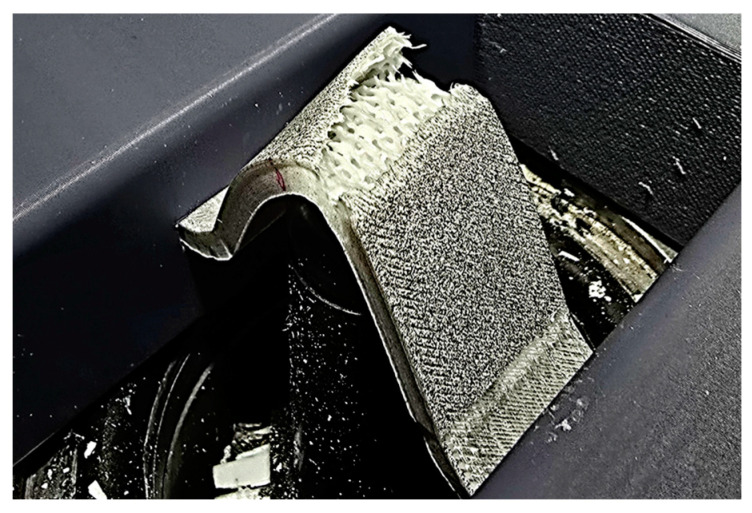
Elongation in the PETG gyroid-filled specimen (i.e., PETG_GyF35%).

A further advantage of the DIC test is that the individual failure states and the deformation of the specimens can be clearly observed. More specific results are shown in Figure 6, Figure 7, Figure 8 and Figure 9.

In Figure 6, it can be seen that the PETG material stretches in the inter-pattern area as a function of the fill, i.e., local stretching occurs at several points, which may also be responsible for better results. This type of phenomenon suggests that the material has a better load-bearing capacity in the patterns, while the material fails faster in the “empty shell”. It can be seen that, although the PETG test specimens achieved similar elongations, the filling density and the pattern had a significant effect on the failure state, so there is an apparent effect on the way the material will fail.

The difference in destruction between the set and laid samples is clearly observed in the case of PLA. The broken specimens clearly show that the set specimen only opened along the layers, while the laid specimen is wholly broken. In Figure 7, it can be seen how the PLA specimens broke.

The carbon-reinforced material showed higher forces but more brittle behavior, as can be seen in Figure 8. The advantage of using this material is that similar mechanical properties can be achieved from relatively few materials compared with other materials.

On the PETG gyroid-filled specimens, it can be seen that there is no localized elongation in this pattern, but the whole specimen follows the shape change together. This behavior is more favorable as the layers move together, making this pattern more suitable for this type of loading. Figure 9 shows one of the PETG gyroid-filled specimens.

**Figure 10:**
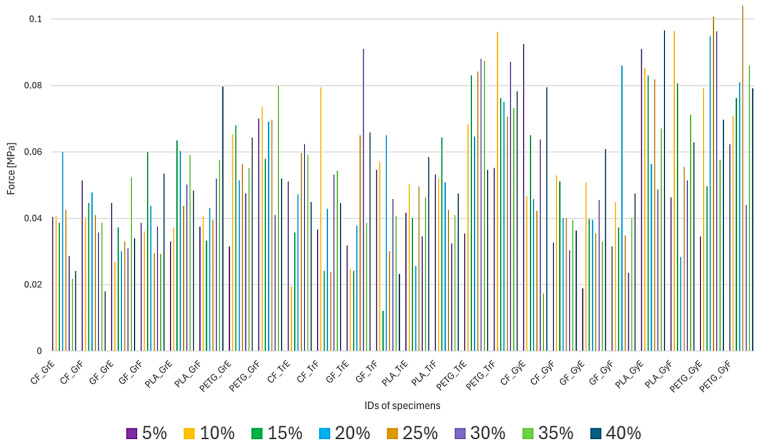
Maximum Force Values of the Specimens.

When examining the force data, it was observed that the values were quite noisy. This is because the equipment used does not have a direct force measuring cell, but the force can be measured from the hydraulic pressure. In the case of fiber-reinforced composite specimens, it could be expected that small forces would occur due to their expected brittleness compared to the capabilities of the equipment, which is in the lower limit range of the hydraulic force gauge. Thus, in the evaluation, the force maxima were examined rather than the full curves because noise does not distort the results as much, and the results for the maxima are shown in Figure 10.

An overview of the results shows that specimens with 20-25-30% infill in most material groups achieved higher forces. When reviewing the materials, PETG, carbon fiber, and glass fiber with gyroid and triangle infill patterns gave the highest forces.

Among the composite materials, the laid arrangement gave better results. It is also interesting to observe that similar force maxima can be obtained for PLA material using a gyroid pattern. For PETG, the gyroid and triangle fill patterns gave better results in both the stationary and laid-down cases.

**Figure 11:**
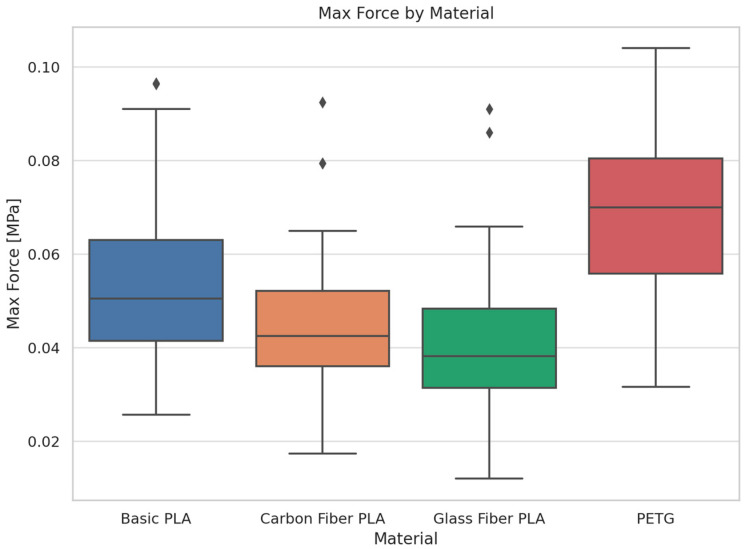
Boxplot of Maximum Force for Various Materials.

The boxplot (Figure 11) shows the statistical distribution of the maximum force values sustained by the tested materials (basic PLA, carbon fiber PLA, glass fiber PLA, and PETG).

Samples made from PETG show the highest maximum force, meaning they withstand the highest load. Basic PLA ranks second in strength, with a broader spread suggesting more variability in performance. Carbon fiber PLA and glass fiber PLA display lower maximum force values. Interestingly, despite being fiber-reinforced, they do not outperform PETG or even basic PLA in strength. Based on the findings of this study, PETG demonstrated the highest load-bearing capacity among the tested materials.

**Figure 12:**
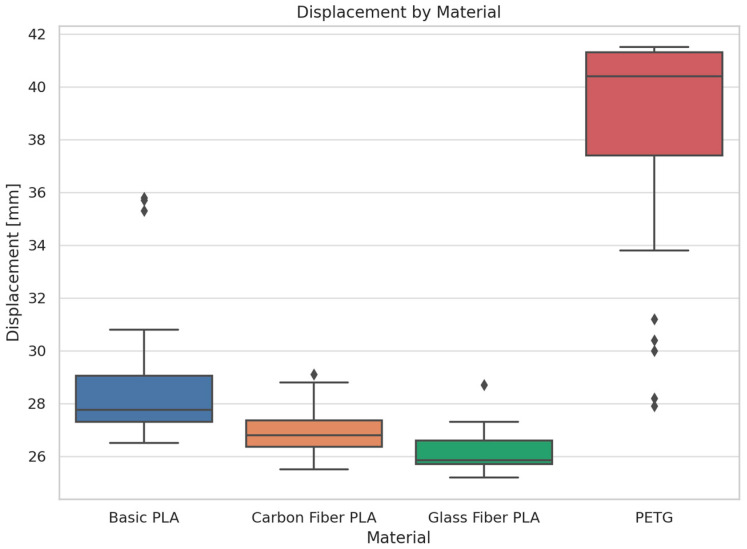
Boxplot of Displacement Across Different Materials.

The boxplot (Figure 12) illustrates the statistical distribution of displacement values for the tested materials (basic PLA, carbon fiber PLA, glass fiber PLA, and PETG). PETG samples exhibited more significant displacement, indicating a more elastic and flexible material. Carbon fiber PLA and glass fiber PLA showed low displacement, meaning they are stiffer. Basic PLA falls somewhere in between—more flexible than the fiber-reinforced variants but less flexible than PETG. In conclusion, PETG combines strength and flexibility, whereas fiber-reinforced PLA materials exhibit greater stiffness but tend to fail under lower loads.

**Figure 13:**
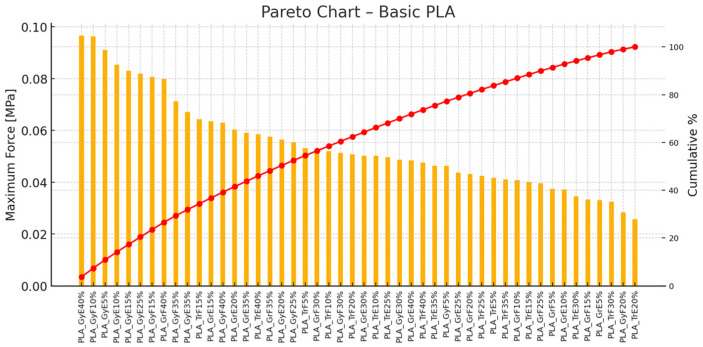
Pareto Chart—Basic PLA (orange color represents the maximum force values, hence red color line means the cumulative percentages).

Figure 13 illustrates a Pareto diagram with the maximum force values [MPa] for various printing settings using basic PLA material. The PLA_GrE20%, PLA_GrE25%, and PLA_GrE30% specimens deliver the best results. The top two account for about 75% of the total force. The trend again shows that 20–30% infill is optimal for basic PLA.

**Figure 14:**
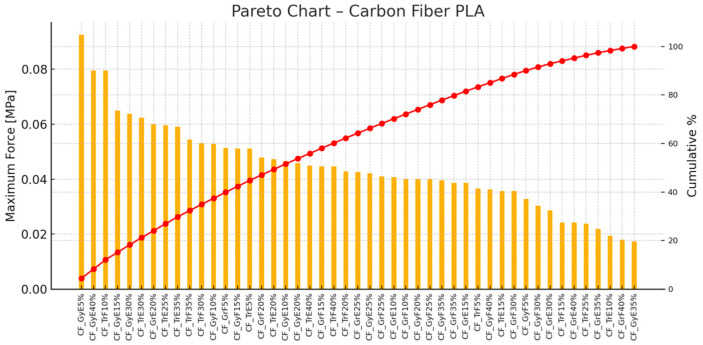
Pareto Chart—PLA Reinforced with Carbon Fiber (orange color represents the maximum force values, hence red color line means the cumulative percentages).

Figure 14 presents a Pareto analysis of carbon fiber PLA printed samples, highlighting the ranked printing settings based on maximum force [MPa]. The chart shows that CF_GrE20% achieved the highest maximum force, followed by CF_GrE25% and CF_GrE30%. According to the red cumulative line, the top three specimens account for more than 80% of the total force. This suggests that the 20–30% infill range provides outstanding mechanical performance for this material.

**Figure 15:**
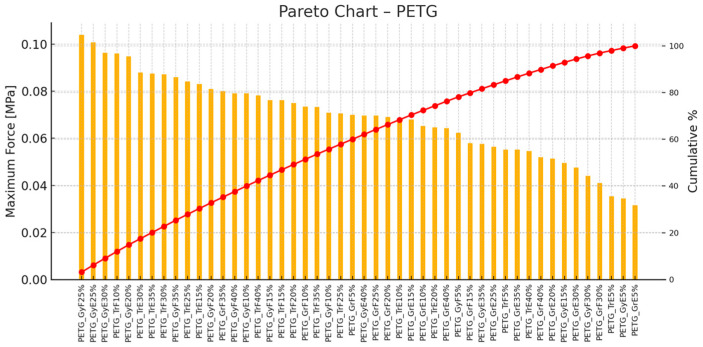
Pareto Chart—PETG (orange color represents the maximum force values, hence red color line means the cumulative percentages).

Figure 15 is a Pareto diagram illustrating the maximum force values [MPa] for various printing settings using PETG material. The most substantial specimen is PETG_GrE25%, followed by PETG_GrE20% and PETG_GrE30%. These three account for about 80% of the total force. For PETG, too, the 20–30% infill range delivers the best mechanical performance.

**Figure 16:**
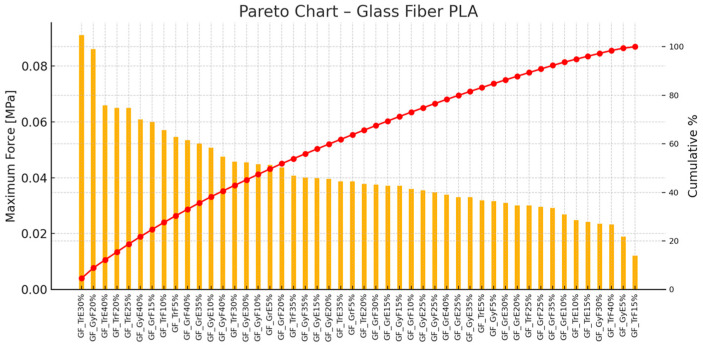
Pareto Chart—PLA Reinforced with Glass Fiber (orange color represents the maximum force values, hence red color line means the cumulative percentages).

Figure 16 shows a Pareto analysis of glass fiber PLA printed samples, highlighting the ranked printing settings based on maximum force [MPa]. The most substantial specimen is GF_GrE20%, followed by GF_GrE25% and GF_GrE30%. Together, these three cover approximately 85% of the total performance. Higher infill percentages prove more beneficial, with values above 20% being particularly effective.

**Figure 17:**
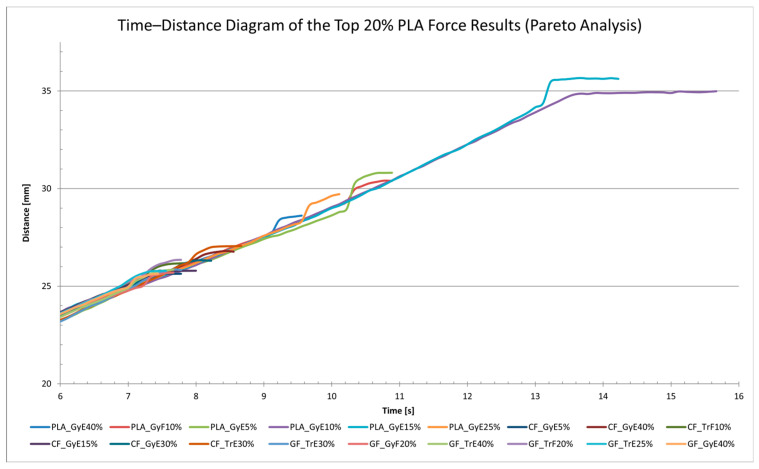
The top 20% PLA force results from Pareto analysis, shown in time-distance.

Figure 17 is the time–distance diagram that visualizes the deformation behavior of the top 20% of 3D printed PLA specimens. The purpose of the analysis is to identify which combinations of material type, infill pattern, printing orientation, and infill percentage performed best under load.

The specimen showing the highest elongation was PLA_GyE15% (35.66 mm), followed by PLA_GyE10% (34.98 mm) and PLA_GyE5% (30.81 mm). All three had gyroid infill and were printed in a vertical (E) orientation, indicating that this combination is particularly favorable for ductility. Interestingly, moderate infill percentages (10–15%) resulted in greater elongation than higher-density configurations such as PLA_GyE40%.

The diagram does not exclusively contain gyroid-patterned specimens; several triangular (Tr) infill samples also made it into the top 20%, particularly among CF (carbon fiber) and GF (glass fiber) materials. This highlights the relevance of the infill pattern, especially when working with high-strength composite materials.

In conclusion, the combination of gyroid infill, vertical orientation, and moderate infill density appears to be optimal for improving the deformation capability of PLA specimens.

## 4. Conclusions

The test results show PETG exhibits stronger and more stable mechanical properties than PLA, especially for grid and gyroid filling patterns, where the measured strength values are generally higher. In the case of PLA, the gyroid pattern initially shows more favorable results, especially at lower infill ratios, but with increasing infill ratios, the strength values start to decrease. It is important to note that increasing the infill ratio does not always result in an apparent increase in strength, so selecting the appropriate pattern and infill percentage is advisable to achieve optimal mechanical properties.

The PETG material was used as a reference in the experiments, which shows good behavior under bending stress. PLA and reinforced PLAs perform worse but are cheaper and can be easily printed with simpler printers.

Although carbon and glass composites provide a more stable structure, their mechanical properties are generally inferior to PLA and PETG. These materials are less sensitive to fill pattern and fill ratio variations, so their strength values are more uniform under different print settings. The tests showed that the carbon fiber and glass fiber PLA filaments behaved similarly; however, regarding both the tolerable force and the deformation achievable, the carbon fiber PLA filament performed slightly better. If bending is the expected stress for a structure, it is preferable to use basic PLA material, which shows better results than the fiber-reinforced filaments.

The experiments also demonstrated that the correct infill pattern and density are needed to achieve these results. The carbon fiber PLA and glass fiber PLA filaments show better results at grid fill patterns and lower fill densities. This may be of practical importance when low fill densities have to be chosen (for weight optimization) and when bending stresses are expected.

The practical significance of the results may be that the tests have shown an optimum infill value for these materials, which is between 20 and 30%, so for load-bearing elements, there is no reason to use higher infill. Further experiments will be conducted to optimize the exact fill rates for materials. The results obtained from this research will be applied to fabricating battery enclosures and other protective storage enclosures.

## Figures and Tables

**Table 1:** Properties of used filaments.

Specific	Basic PLA	PETG	Carbon Fiber PLA	Glass Fiber PLA
Nozzle [°C]	190–210	235–250	190–230	190–230
Bed [°C]	50–60	80–95	45–60	45–60
Layer height [mm]	0.4	0.4–1.0	0.4	0.4
Max speed [mm/s]	30–70	60	50–300	40–100
Color	Blue	White	Green	Natural

**Table 2:** Physical and mechanical properties.

Specific	Basic PLA	PETG	Carbon Fiber PLA	Glass Fiber PLA
Density [g/cm^3^]	1.24	1.27	1.21	1.31
Melt Flow Index [g/10 min]	6 (210 °C/2.16kg)	-	5.37 (190 °C/2.16 kg)	6.36 (190 °C/2.16 kg)
Tensile Strength [MPa]	60	50	39	59.27
Elongation at Break [%]	6	120	4.27	7.99
Flexural Strength [MPa]	83	71	103	85.01
Flexural Modulus [MPa]	3800	2150	5003	4414.89
IZOD Impact Strength	16 [J/m]	85 [J/m]	5.08 [kJ/m^2^]	10.16 [kJ/m^2^]

**Table 3:** Properties of used 3D printers.

Specific	Bambu Lab X1 Carbon	Bambu Lab A1 Mini
Manufacturer (city, country)	Bambu Lab (Shenzhen, China)	
Nozzle size [mm]	0.4	0.4
Extruder system	Direct drive	Direct drive
Filament diam.	1.75 mm	1.75 mm
Hotend	All metal	All metal
Bed leveling	Dual auto	Automatic

**Table 4:** Experimental matrix (1—PLA, 2—PETG, 3—Carbon Fiber PLA, 4—Glass Fiber PLA). The symbols “+” mean that the related tests were executed during the experiments of the current research.

Infill Percentage [%]	Grid (Gr)								Triangle (Tr)								Gyroid (Gy)							
	On-Edge (E)				Flat (F)				On-Edge (E)				Flat (F)				On-Edge (E)				Flat (F)			
	1	2	3	4	1	2	3	4	1	2	3	4	1	2	3	4	1	2	3	4	1	2	3	4
5	+	+	+	+	+	+	+	+	+	+	+	+	+	+	+	+	+	+	+	+	+	+	+	+
10	+	+	+	+	+	+	+	+	+	+	+	+	+	+	+	+	+	+	+	+	+	+	+	+
15	+	+	+	+	+	+	+	+	+	+	+	+	+	+	+	+	+	+	+	+	+	+	+	+
20	+	+	+	+	+	+	+	+	+	+	+	+	+	+	+	+	+	+	+	+	+	+	+	+
25	+	+	+	+	+	+	+	+	+	+	+	+	+	+	+	+	+	+	+	+	+	+	+	+
30	+	+	+	+	+	+	+	+	+	+	+	+	+	+	+	+	+	+	+	+	+	+	+	+
35	+	+	+	+	+	+	+	+	+	+	+	+	+	+	+	+	+	+	+	+	+	+	+	+
40	+	+	+	+	+	+	+	+	+	+	+	+	+	+	+	+	+	+	+	+	+	+	+	+

**Table 5:** Result of displacement.

**PLA**	**Grid (Gr)**		**Triangle (Tr)**		**Gyroid (Gy)**		**PETG**	**Grid (Gr)**		**Triangle (Tr)**		**Gyroid (Gy)**	
**%**	**On-Edge** **(E)**	**Flat** **(F)**	**On-Edge** **(E)**	**Flat** **(F)**	**On-Edge** **(E)**	**Flat** **(F)**	**%**	**On-Edge** **(E)**	**Flat** **(F)**	**On-Edge** **(E)**	**Flat** **(F)**	**On-Edge** **(E)**	**Flat** **(F)**
5	27.5	27.3	26.7	28.1	30.8	30.6	5.0	40.1	27.9	28.2	31.2	30.0	41.3
10	26.8	27.4	26.5	28.7	35.3	30.6	10.0	40.6	41.0	41.5	41.0	41.2	41.2
15	29.3	27.3	28.3	27.9	35.7	29.8	15.0	37.3	37.6	41.3	41.3	30.4	41.4
20	29.0	26.8	27.3	27.8	35.8	27.7	20.0	40.1	39.8	41.3	39.6	41.3	40.5
25	27.9	27.3	27.1	27.3	29.7	27.7	25.0	40.1	37.2	41.3	34.7	41.3	40.4
30	29.2	27.1	27.7	27.4	29.0	27.3	30.0	40.2	41.4	41.0	37.3	41.4	33.8
35	29.5	27.4	27.9	27.4	28.3	27.5	35.0	40.5	41.0	41.0	40.0	41.3	37.5
40	29.7	27.5	28.3	27.5	28.5	27.1	40.0	40.6	40.1	36.7	36.3	41.3	37.8
**GLASS**	**Grid (Gr)**		**Triangle (Tr)**		**Gyroid (Gy)**		**CARBON**	**Grid (Gr)**		**Triangle (Tr)**		**Gyroid (Gy)**	
**%**	**On-Edge** **(E)**	**Flat** **(F)**	**On-Edge** **(E)**	**Flat** **(F)**	**On-Edge** **(E)**	**Flat** **(F)**	**%**	**On-Edge** **(E)**	**Flat** **(F)**	**On-Edge** **(E)**	**Flat** **(F)**	**On-Edge** **(E)**	**Flat** **(F)**
5	25.6	28.7	25.6	26.9	25.7	26.0	5.0	25.5	27.1	26.1	26.8	25.8	27.5
10	25.4	27.3	25.8	27.0	25.8	25.9	10.0	25.5	26.8	25.9	26.8	25.6	27.8
15	25.4	26.8	25.8	26.7	25.2	25.8	15.0	26.0	26.7	26.4	26.6	26.1	27.4
20	25.8	26.9	26.2	26.8	25.2	25.7	20.0	26.5	28.5	26.1	26.8	25.7	27.5
25	25.9	26.2	26.1	26.6	25.2	25.7	25.0	27.1	26.9	26.8	26.7	25.8	27.2
30	25.7	26.5	25.9	26.6	25.6	25.8	30.0	27.8	27.3	27.1	26.8	26.4	27.4
35	25.7	26.4	26.1	26.9	25.7	25.8	35.0	29.1	27.5	27.5	26.3	26.0	27.0
40	25.6	26.7	26.0	26.7	25.7	25.8	40.0	28.7	28.8	26.8	27.1	26.8	27.1

## Data Availability

All of the data are within the paper.

## List of Abbreviations

3D	Three-Dimension or Three-Dimensional
AM	Additive Manufacturing
CF	Carbon Fiber
DIC	Digital Image Correlation
ECT	Erichsen Cupping Test
FDM	Fused Deposition Modeling
GF	Glass Fiber
LOM	Laminated Object Manufacturing
PA	Polyamide
PETG	Polyethylene Terephthalate Glycol
PLA	Polylactic Acid
SLA	Stereolithography
SLS	Selective Laser Sintering
